# Single-Shot Ultrasound-Guided Transversus Abdominis Plane Block Versus Intravenous Patient-Controlled Analgesia for Early Recovery After Laparoscopic Cholecystectomy: A Retrospective Cohort Study

**DOI:** 10.3390/jcm15031120

**Published:** 2026-01-31

**Authors:** Youngjoo Park

**Affiliations:** Department of Anesthesiology and Pain Medicine, Samsung Changwon Hospital, College of Medicine, Sungkyunkwan University, Changwon 51353, Republic of Korea; anesun12345@naver.com

**Keywords:** laparoscopic cholecystectomy, transversus abdominis plane block, intravenous patient-controlled analgesia, postoperative analgesia, enhanced recovery, opioid-free analgesia

## Abstract

**Background:** Effective postoperative analgesia after laparoscopic cholecystectomy (LC) should facilitate rapid recovery while minimizing exposure to opioid-related adverse events, a central goal of enhanced recovery after surgery (ERAS). Although intravenous patient controlled analgesia (IV-PCA) remains widely used, its gastrointestinal and mobilization-impairing side effects may hinder early recovery. **Methods:** This retrospective cohort study included adult patients who underwent elective laparoscopic cholecystectomy, all performed using a standardized three-port technique, between January 2025 and December 2025. Patients with conversion to open surgery, concurrent procedures, incomplete medical records, or American Society of Anesthesiologists physical status ≥ IV were excluded. Patients received either a single-shot ultrasound-guided subcostal transversus abdominis plane (TAP) block with 0.19% ropivacaine or conventional fentanyl-based IV-PCA. Postoperative analgesic requirements, functional recovery outcomes, and safety profiles were evaluated. **Results:** All patients in the Group TAP (n = 60) required no rescue analgesia during the first 12 postoperative hours and did not require nonsteroidal anti-inflammatory drugs or IV-PCA within 24 h. Early recovery milestones were consistently achieved, including preserved early ambulation, prompt tolerance of oral intake, and smooth transition to oral acetaminophen 650 mg orally three times daily from postoperative day 1. All Group TAP patients met the discharge criteria by postoperative day 2 without opioid-related adverse events or signs of local anesthetic systemic toxicity. In contrast, the Group IV-PCA (n = 60) exhibited a high incidence of opioid-related adverse effects, frequent PCA interruption or discontinuation, delayed functional recovery, and prolonged hospitalization. **Conclusions:** A single-shot ultrasound-guided subcostal TAP block using low-concentration ropivacaine can function as a reliable, opioid-free primary analgesic strategy after laparoscopic cholecystectomy, effectively supporting ERAS-consistent early recovery. This approach represents a practical and clinically meaningful alternative to conventional IV-PCA in routine LC.

## 1. Introduction

Laparoscopic cholecystectomy (LC) is the gold-standard treatment for benign gallbladder disease, offering minimal surgical trauma, reduced tissue injury, and lower perioperative stress compared with open surgery.

These attributes make LC particularly suitable for application of enhanced recovery after surgery (ERAS) protocols [1]. Despite minimally invasive techniques, many patients still experience moderate postoperative pain that delays ambulation and discharge. Intravenous patient-controlled analgesia (IV-PCA) remains a standard approach but is frequently limited by nausea, vomiting, dizziness, and headache, often leading to early discontinuation and increased rescue analgesic use [2,3,4,5,6,7].

The transversus abdominis plane (TAP) block has emerged as a simple, ultrasound-guided regional technique that provides somatic pain relief with minimal systemic effects. Previous studies have demonstrated its benefits in various abdominal procedures [8,9,10,11,12], but comparative data versus IV-PCA focusing on rescue analgesic frequency and recovery quality remain limited.

This retrospective comparative cohort study aimed to evaluate whether a single-shot ultrasound-guided transversus abdominis plane (TAP) block could serve as the primary postoperative analgesic strategy compared with intravenous patient-controlled analgesia after elective laparoscopic cholecystectomy, with a focus on early recovery outcomes.

## 2. Materials and Methods

### 2.1. Study Design and Ethics

This was a single-center, retrospective comparative study involving the review of electronic medical records (EMRs) of patients who underwent elective laparoscopic cholecystectomy (LC) between January 2025 and December 2025. The study was approved by the Institutional Review Board (IRB) of Samsung Changwon Medical Center (SCMC IRB-2025-11-001) and conducted in accordance with the Declaration of Helsinki. The requirement for informed consent was waived due to the retrospective nature of the study.

### 2.2. Patient Selection and Group Allocation

Group allocation was non-randomized and was determined during standardized preoperative counseling by the attending anesthesiologist, followed by patient acceptance of the proposed analgesic strategy. This pragmatic approach reflects real-world decision-making; however, it may introduce confounding by indication, which we addressed using multivariable adjustment and sensitivity analyses (see Statistical Analysis). In our institution during the study period, both analgesic pathways (single-shot ultrasound-guided subcostal TAP block and conventional IV PCA) were consistently available as part of routine postoperative pain management options for laparoscopic cholecystectomy. There were no logistical, scheduling, or resource constraints that systematically limited access to TAP blocks, and availability was not preferentially restricted to specific operating rooms or surgical teams. The choice of analgesic strategy was therefore based on shared decision-making reflecting patient preference and clinical recommendation, rather than limited by institutional infrastructure or personnel availability. To minimize operator-dependent variability, all TAP blocks were performed by a single experienced anesthesiologist according to a standardized technique.

A total of 120 adult patients (≥18 years old) who underwent elective LC for symptomatic cholelithiasis were included. Patients were categorized into two groups based on the primary postoperative analgesia strategy employed:

1. Group TAP (n = 60): Patients who received a single-shot, ultrasound-guided transversus abdominis plane (TAP) block as part of a multimodal analgesia protocol.
2. Group IV-PCA (n = 60): Patients who received conventional systemic postoperative analgesia via intravenous patient-controlled analgesia (IV-PCA).

Inclusion criteria were: ASA physical status I–III, age 18–75 years, and undergoing elective LC. Exclusion criteria were emergency surgery, intraoperative conversion to open cholecystectomy, combined procedures, incomplete medical records, and ASA physical status ≥ IV. Group allocation was non-randomized and was determined by the attending anesthesiologist’s clinical recommendation during standardized preoperative counseling, followed by patient acceptance of the proposed analgesic strategy.

### 2.3. Anesthesia and Analgesia Protocols

All patients received standardized general anesthesia and intraoperative opioid use followed an institutional standardized protocol and did not differ materially between groups. Intraoperative opioid administration (agent and total dose) during induction and maintenance of anesthesia was extracted from the anesthesia record for all patients and compared between groups. This variable was additionally considered in adjusted analyses to reduce confounding when interpreting postoperative adverse events. Postoperative analgesic management differed between groups as follows.

#### 2.3.1. Group TAP (ERAS-Compliant Multimodal Analgesia)

**Figure 1:**
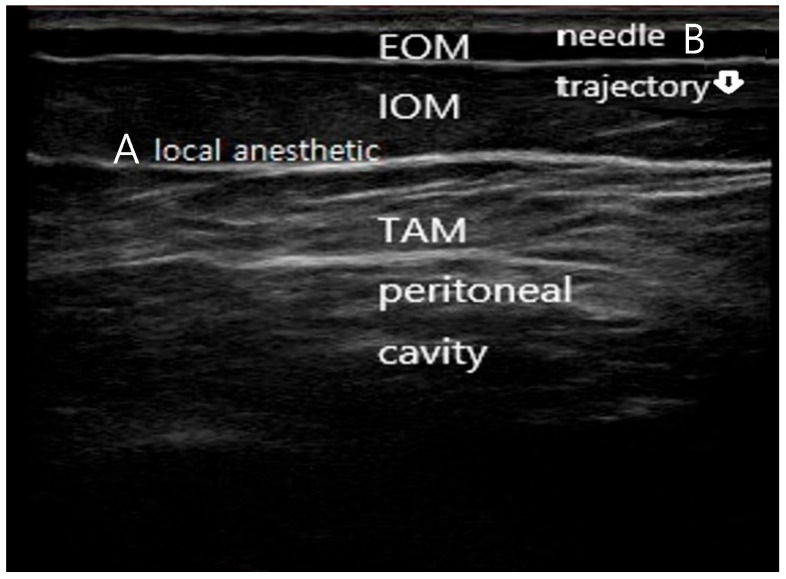
Ultrasound-guided subcostal transversus abdominis plane (TAP) block technique. (A) Ultrasound image demonstrating the subcostal abdominal wall anatomy, including the external oblique muscle (EOM), internal oblique muscle (IOM), transversus abdominis muscle (TAM), and peritoneal cavity. The target fascial plane between the internal oblique and transversus abdominis muscles is indicated. (B) In-plane needle advancement with a near-vertical trajectory toward the subcostal TAP plane under real-time ultrasound guidance, with hydrodissection confirming correct deposition of local anesthetic within the target fascial plane. The needle is faintly visualized advancing in-plane toward the target fascial plane, consistent with real-time ultrasound-guided subcostal TAP block technique.

Patients received a single-shot, ultrasound-guided bilateral subcostal transversus abdominis plane (TAP) block (Figure 1), performed by a single experienced anesthesiologist approximately 10 min before the end of surgery. The ultrasound probe was positioned just inferior to the subcostal margin with slight anterior angulation to optimize visualization of the internal oblique–transversus abdominis fascial plane. Using high-resolution real-time ultrasound guidance (13 MHz), the needle was introduced in-plane with a near-vertical trajectory. Minor anterior adjustments of probe tilt were made as needed to maintain continuous visualization of the needle tip. Local anesthetic was injected precisely into the target fascial plane, with clear hydrodissection confirming correct placement.

A total of 20 mL of 0.19% ropivacaine was injected bilaterally. No basal opioid analgesia (IV-PCA) was initiated. Scheduled oral acetaminophen 650 mg three times daily (TID) was prescribed starting on postoperative day (POD) 1 following initiation of oral intake.

#### 2.3.2. Group IV-PCA (Conventional Analgesia)

Patients received intravenous patient-controlled analgesia (IV-PCA) initiated 10 min before the end of surgery. The PCA solution contained fentanyl (total dose 500–1000 μg according to institutional standard and patient risk, diluted in 0.9% saline to a total volume of 100 mL, delivered at a basal rate of 1 mL/h with a bolus dose of 1 mL and a lockout interval of 8 min).

### 2.4. Postoperative Management and Outcome Measures

Both groups followed a standardized postoperative care pathway that included early ambulation and a scheduled diet advancement. Oral intake (clear liquids) was initiated in the evening of surgery (approximately 6 h postoperatively) and advanced to a soft diet on the morning of postoperative day (POD) 1, coinciding with initiation of oral acetaminophen 650 mg.

To evaluate the influence of the initial analgesic technique on early recovery, two predefined assessment time points were established:

Time A, initiation of the primary analgesic method (immediately after surgery, prior to extubation);

Time B, arrival at the general ward (approximately 40–60 min after Time A).

The first postoperative Numerical Rating Scale (NRS) pain score was recorded at Time B.

#### 2.4.1. Primary Outcome

The primary outcome was postoperative length of stay (LOS), defined as the interval from the end of surgery to discharge readiness.

#### 2.4.2. Secondary Outcomes

Secondary outcomes included measures of functional recovery and opioid-related adverse events.

Postoperative adverse events, including nausea, vomiting, dizziness, headache, urinary retention, and delayed passage of flatus, were assessed using identical chart-based definitions in both groups. Events were retrospectively captured from standardized postoperative nursing records and physician progress notes during the first 24 postoperative hours. To minimize detection bias, the same assessment time window and documentation framework were applied to both groups.

(1) *Time to first rescue analgesic*, defined as the interval from Time A to the first administration of supplementary analgesia other than scheduled oral medication.
(2) *Opioid-induced side effects (OISEs)* and *analgesic discontinuation* including nausea, vomiting, delayed passage of flatus, and failure of spontaneous voiding requiring bladder catheterization. Discontinuation of IV-PCA was defined as cessation of PCA delivery due to intolerable opioid-related adverse effects, as documented in the medical record.
(3) *Functional recovery markers*, including time to tolerance of a soft or regular diet, time to first flatus, and the proportion of patients meeting discharge criteria by POD 2.

#### 2.4.3. Outcome Definitions

Discharge readiness was defined a priori according to institutional ERAS-based criteria, including adequate pain control with oral analgesics alone, tolerance of oral intake without nausea or vomiting, independent ambulation, stable vital signs, spontaneous voiding, and absence of postoperative complications requiring inpatient management. Patients were considered discharge-ready once all criteria were met, independent of administrative discharge timing. Discharge readiness was assessed daily at [specify time, e.g., 8:00 AM] during morning rounds. The attending surgical team and senior ward nurses utilized a standardized institutional ERAS-based checklist aligned with internationally accepted ERAS principles. Patient-reported outcomes for pain were measured using the Visual Analog Scale (VAS), and functional milestones (e.g., ambulation, bowel function) were cross-verified with nursing documentation to ensure objective assessment.

### 2.5. Statistical Analysis

Continuous variables are presented as mean ± standard deviation and categorical variables as number (%). Between-group comparisons were performed using the independent-samples *t*-test or the Mann–Whitney U test for continuous variables, and the chi-square test or Fisher’s exact test for categorical variables, as appropriate.

Because allocation to Group TAP block or Group IV-PCA was non-randomized and primarily influenced by the attending anesthesiologist’s pre-anesthetic counseling, potential selection bias and confounding were anticipated. To account for this, multivariable regression analyses were conducted for key outcomes, with analgesic modality (TAP vs. IV-PCA) as the main exposure. Prespecified covariates included age, sex, ASA physical status, history of postoperative nausea and vomiting or motion sickness, chronic opioid or sedative use, severity of gallbladder pathology (chronic vs. acute/complicated cholecystitis), and the anesthesiologist responsible for preoperative counseling.

Linear regression was used for continuous outcomes, and logistic regression for binary outcomes. Adjusted estimates are reported as mean differences or odds ratios with 95% confidence intervals. A two-sided *p* value < 0.05 was considered statistically significant.

All analyses were performed using SPSS Statistics (IBM Corp., Armonk, NY, USA, version 27).

To further mitigate confounding by indication inherent to the non-randomized study design, a propensity score-based sensitivity analysis was additionally performed. Propensity scores were estimated using logistic regression incorporating prespecified baseline covariates, including age, sex, body mass index, ASA physical status, history of postoperative nausea and vomiting, and gallbladder pathology. Inverse probability of treatment weighting (IPTW) was applied to generate a weighted pseudo-population with improved baseline balance between groups, which was assessed using standardized mean differences. Propensity score analyses were conducted using R software (version 4.5.1.; R Foundation for Statistical Computing, Vienna, Austria). Because certain baseline characteristics—particularly history of postoperative nausea and vomiting and disease severity—were strongly associated with treatment allocation in routine clinical practice, some residual imbalance might persist even after IPTW. To ensure the stability and validity of treatment effect estimates, all primary analyses were performed using a doubly robust approach, combining IPTW weights with multivariable regression adjustment. Covariate balance before and after inverse probability of treatment weighting (IPTW) is presented in Supplementary Table S1.

### 2.6. Artificial Intelligence Statement

No artificial intelligence-assisted technologies were used in the conduct of this study, including study design, data analysis, or manuscript preparation.

## 3. Results

### 3.1. Patient Characteristics

A total of 120 patients who underwent laparoscopic cholecystectomy were included (Group TAP, n = 60; Group IV-PCA, n = 60). Baseline demographic and perioperative characteristics were comparable between groups with respect to age, sex distribution, BMI, and baseline clinical characteristics (Table 1). Although SBP and DBP were slightly higher in the IV-PCA group, this difference did not influence postoperative outcomes, and all patients were classified as ASA I–III. After IPTW, baseline covariates were well balanced between groups (Supplementary Table S1).

**Figure 2:**
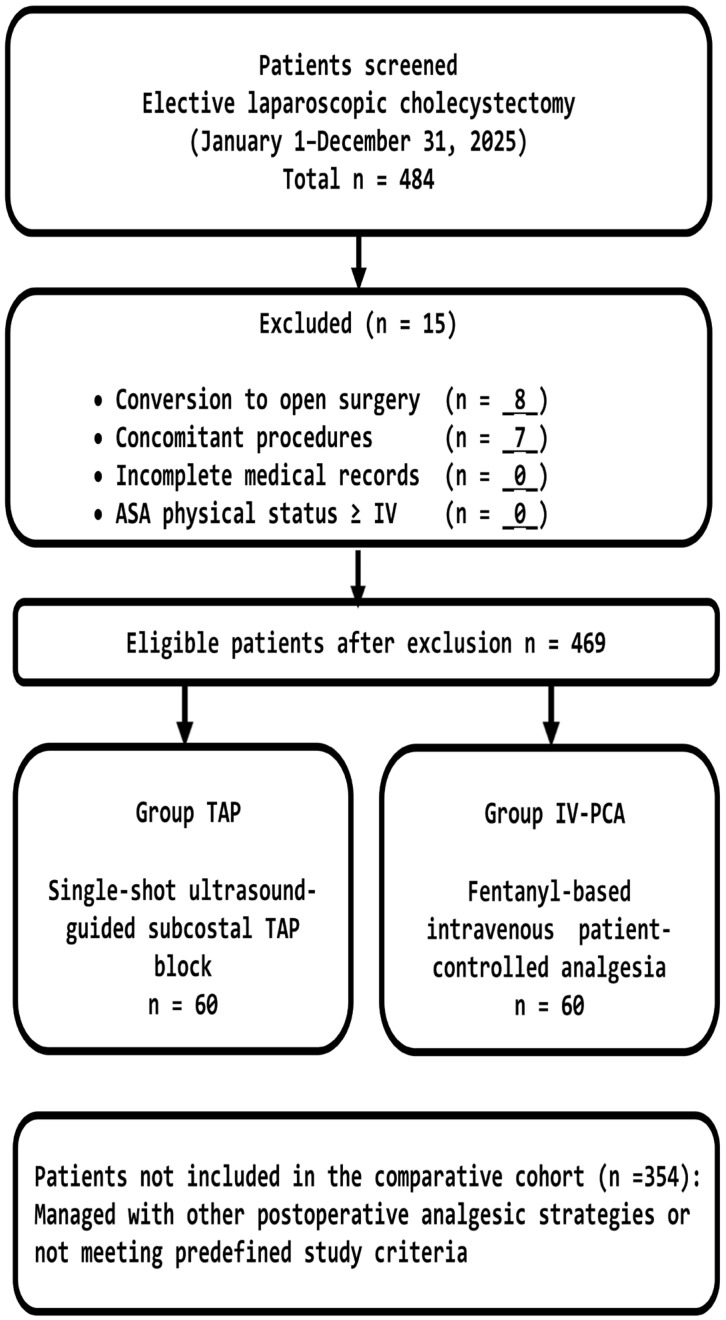
Flow diagram illustrating patient screening, exclusion criteria, and allocation to the TAP block and IV-PCA groups. Group allocation was determined by anesthesiologist counseling and patient preference, reflecting real-world clinical practice.

A patient flow diagram summarizing screening, exclusions with reasons, and final group allocation is provided in Figure 2.

IPTW substantially improved covariate balance across most baseline variables, with standardized mean differences decreasing from moderate imbalance at baseline to <0.15 for age, BMI, and ASA class after weighting. Residual imbalance remained for disease severity and history of postoperative nausea and vomiting, reflecting their intrinsic association with real-world treatment selection, a limitation addressed through additional multivariable adjustment.

### 3.2. Functional Recovery Outcomes

Patients receiving TAP block demonstrated significantly accelerated recovery across all predefined ERAS endpoints (Table 2). Patients in Group TAP achieved significantly faster functional recovery across all predefined ERAS-related endpoints compared with those in Group IV-PCA (Table 2). The time to first ambulation was markedly shorter in patients in Group TAP (3.5 ± 1.2 h) than in those in Group IV-PCA (12.7 ± 4.5 h; *p* < 0.001), representing a large effect size (Cohen’s d = 2.83). Similarly, tolerance of oral intake occurred substantially earlier in Group TAP (5.8 ± 2.1 h vs. 16.3 ± 5.5 h; *p* < 0.001; d = 2.39).

Postoperative pain control at 24 h was significantly superior in Group TAP, with lower NRS pain scores compared with Group IV-PCA (2.2 ± 1.1 vs. 5.8 ± 1.5; *p* < 0.001; d = 2.74). Consistent with this finding, none of the patients in Group TAP required rescue analgesics during the first 24 postoperative hours, whereas 75% of patients in Group IV-PCA required supplemental analgesia (0% vs. 75%; *p* < 0.001; relative risk estimated with a continuity correction = 0.09).

Analgesic regimens on postoperative day 1 also differed substantially between groups. All patients in Group TAP were managed exclusively with oral acetaminophen (650 mg, three times daily), whereas patients in Group IV-PCA frequently required additional non-opioid analgesics in conjunction with acetaminophen.

Early discharge readiness was achieved more frequently in Group TAP, with all patients (100%) meeting discharge criteria by postoperative day 2, compared with only 32% of patients in Group IV-PCA (*p* < 0.001; relative risk = 3.15). Correspondingly, postoperative length of stay was significantly shorter in Group TAP, with a median of 2.0 days (IQR 2–2) versus 5.0 days (IQR 4–5) in Group IV-PCA (*p* < 0.001). Although LOS is summarized using medians, effect size estimates (Cohen’s d = 2.87) were calculated based on the underlying continuous data, indicating a large between-group difference in recovery duration.

Analgesia (single-shot TAP block or IV-PCA) was initiated 10 min before the end of surgery. Pain intensity was first assessed on arrival to the general ward (approx. 70 min postoperatively); no significant difference in the initial NRS score was observed. All patients in the TAP group demonstrated preserved early physiological recovery (spontaneous voiding, return of bowel function, and tolerance of clear liquids) within 8 h postoperatively without requiring rescue analgesics. In contrast, the IV-PCA group experienced significant opioid-related delays in functional recovery, leading to prolonged hospitalization. Length of stay (LOS) is presented as median (interquartile range, IQR). IPTW-adjusted analyses for key clinical outcomes are summarized in Supplementary Table S2.

### 3.3. Opioid-Related Adverse Events and PCA Management

Opioid-related adverse events occurred frequently and early in the IV-PCA group (Table 3). Overall, 60% of patients receiving IV-PCA experienced at least one clinically significant opioid-related adverse effect, most commonly headache, nausea/vomiting, and dizziness, while no patient in the TAP group reported any adverse event (all *p* < 0.001).

These adverse effects translated directly into disruption of the planned analgesic regimen. More than half of IV-PCA patients (53.3%) required temporary PCA clamping on the day of surgery, and one-quarter experienced recurrent adverse events necessitating repeated interventions. PCA intolerance progressed rapidly, with the first clamping occurring at a mean of 11.6 h after initiation, and permanent discontinuation of IV-PCA occurring in 70% of patients within 48 h (Table 4). In contrast, no TAP patient required analgesic interruption, rescue antiemetics, or protocol modification.

Importantly, the need for PCA interruption and early discontinuation was driven by opioid intolerance rather than inadequate analgesia, as reflected by the high incidence of nausea, vomiting, dizziness, and headache. These findings highlight a fundamental instability of opioid-based PCA in the immediate postoperative period and underscore its incompatibility with ERAS-oriented recovery after laparoscopic cholecystectomy.

Notably, the high incidence of opioid-related adverse events in the IV-PCA cohort was not attributable to excessive opioid dosing. Stratified analysis according to total fentanyl dose categories (low < 600 μg, moderate 600–799 μg, and high ≥ 800 μg) revealed no significant differences in the incidence of headache, nausea/vomiting, or dizziness. This finding suggests that clinically relevant opioid intolerance occurred independently of PCA dose category, indicating an inherent vulnerability to opioid-related adverse effects rather than a dose-dependent phenomenon in the immediate postoperative period. Operational definitions and assessment frameworks for adverse events and discharge readiness are detailed in Supplementary Table S3.

### 3.4. Dose Stratification and Temporal Pattern of Adverse Events

Consistent with this observation, opioid-related adverse events manifested early after surgery, with a rapid temporal pattern of PCA disruption. More than half of patients receiving IV-PCA required temporary clamping on the day of surgery, and the mean time from PCA initiation to first intervention was approximately 12 h. Permanent discontinuation of IV-PCA frequently followed within the first 48 postoperative hours. Together, these findings demonstrate that opioid-related adverse effects emerged early and unpredictably, regardless of fentanyl dose category, underscoring the inherent instability of opioid-based PCA during the critical early recovery phase after laparoscopic cholecystectomy.

### 3.5. Summary of Comparative Outcomes

Overall, TAP block provided superior recovery, complete elimination of opioid-related adverse events, markedly reduced opioid requirements, and significantly shorter postoperative hospital stay compared with IV-PCA. The magnitude of these differences, particularly effect sizes exceeding 2.0 across multiple endpoints, supports the clinical superiority and ERAS-aligned benefit of TAP block as a primary analgesic strategy.

## 4. Discussion

The core finding of this retrospective comparative study is that a single-shot ultrasound-guided subcostal Transversus Abdominis Plane (TAP) block can function as the sole primary analgesic strategy after laparoscopic cholecystectomy (LC), reliably supporting an Enhanced Recovery After Surgery (ERAS) pathway without the need for intravenous patient-controlled analgesia (IV-PCA). ERAS programs emphasize minimization of systemic opioid exposure because opioids impair physiologic recovery across multiple domains, including gastrointestinal motility, detrusor function, and cognitive performance [1,2,3,8]. Although this principle is well established, few clinical studies have quantified how rapidly IV-PCA disrupts early ERAS milestones in a real-world LC cohort.

In the present study, administration of 0.19% ropivacaine as a single subcostal TAP block provided effective analgesia during the first 24 postoperative hours, without the need for rescue intravenous opioids or nonsteroidal anti-inflammatory drugs, and allowed a smooth transition to oral acetaminophen from postoperative day (POD) 1. Clinically, this translated into preserved early mobilization, uninterrupted oral intake, and universal discharge readiness by POD 2. Specifically, the TAP group demonstrated a markedly shorter time to first ambulation (3.5 ± 1.2 h vs. 12.7 ± 4.5 h; *p* < 0.001) and earlier oral intake tolerance (5.8 ± 1.2 h vs. 16.3 ± 5.5 h; *p* < 0.001) compared to the IV-PCA group. While the retrospective nature of this study precludes a definitive causal claim, these observations suggest a potential association between the opioid-sparing effect of TAP block and the achievement of early functional recovery. By limiting opioid-related side effects such as sedation and dizziness, the TAP strategy may contribute to more efficient progress toward key ERAS milestones in this clinical cohort. In contrast, patients managed with fentanyl-based IV-PCA experienced a high burden of opioid-induced side effects and frequent protocol failure during the critical early postoperative period, often necessitating premature PCA discontinuation and undermining ERAS recovery targets. These findings indicate that an opioid-free TAP strategy can serve not merely as an adjunct, but as a central analgesic component in fast-track LC. Importantly, the non-randomized, clinician-guided allocation in this study reflects real-world decision-making in contemporary perioperative practice, thereby enhancing the external validity and practical relevance of these findings.

Multiple randomized trials and meta-analyses have already established TAP block as an effective opioid-sparing technique in LC [6,7,8,9,10,11,12]. Ultrasound-guided bilateral TAP block reduces intraoperative opioid requirements and postoperative morphine consumption while maintaining technical feasibility and safety [4,6]. In alignment with this robust evidence, recent studies continue to support the integration of TAP blocks into postoperative care. For instance, Dai et al. (2022) reported superior pain management and faster recovery outcomes when TAP block was utilized within clinical protocols for LC [13]. Additionally, a recent meta-analysis by Zhu and Sun (2023) underscores the potential for further enhancing the block’s quality and duration through pharmacological adjuvants, such as dexmedetomidine [14].

Prior work has demonstrated that subcostal TAP block lowers pain scores at rest and during coughing, reduces opioid use, and facilitates early discharge after LC [8,12]. The present study extends this literature in two important ways. First, within a standardized ERAS-like pathway, we demonstrate that single-shot TAP alone—followed only by scheduled oral acetaminophen once bowel function returns—can completely eliminate the need for IV-PCA in an entire LC cohort. Second, this regional-only strategy was sufficiently robust to maintain 100% POD 2 discharge, suggesting that TAP block can act as a true ERAS enabler rather than a supplementary analgesic technique.

The analgesic profile observed in our cohort is consistent with mechanistic data indicating that early post-LC pain is dominated by somatic input from supra-umbilical trocar sites and the upper abdominal wall [15,16,17]. Sensory-mapping studies have shown that subcostal TAP block produces a more cephalad dermatomal spread than posterior approaches, making it particularly suitable for upper abdominal incisions [18]. Randomized and observer-blinded trials comparing subcostal or oblique subcostal TAP techniques with conventional infiltration or lateral TAP approaches have consistently demonstrated superior pain control, reduced opioid consumption, and faster recovery after LC [13,19,20,21]. Collectively, these findings support the mechanistic rationale for preferential use of subcostal TAP block when supra-umbilical somatic nociception is the dominant pain generator.

Recent mechanistic studies provide a contemporary physiological framework that may extend beyond the conventional interpretation of injectate spread visualized on ultrasound. Experimental and cadaveric investigations have demonstrated that local anesthetics such as bupivacaine can traverse anisotropic tissue planes and permeate fascial barriers via diffusion, even in the absence of clearly visible tracer distribution. These findings suggest that the clinical analgesic effects of interfascial plane blocks, including ultrasound-guided TAP blocks, may reflect both macroscopic fascial plane targeting and micro-diffusional spread of the anesthetic agent across adjacent tissues. Such mechanisms could contribute to the extent and variability of postoperative analgesia observed in clinical practice and offer physiological plausibility for the favorable outcomes seen with low-concentration ropivacaine in this cohort. Incorporating these insights underscores the complex pharmacodynamics of TAP blocks and aligns our clinical observations with emerging evidence from preclinical research [22]. In this context, although subcostal TAP block demonstrated clear clinical effectiveness in this study, the mechanisms underlying interfascial plane block analgesia are increasingly recognized as complex and heterogeneous. Experimental and cadaveric evidence suggests that analgesic effects may result not only from visible injectate spread, but also from anisotropic diffusion and trans-fascial permeation, contributing to interindividual variability. This perspective supports cautious interpretation of the predictability of TAP block analgesia across patients.

Despite these advantages, postoperative analgesia after LC has traditionally relied on systemic opioids delivered via IV-PCA. This approach is increasingly recognized as misaligned with ERAS principles, which advocate multimodal, largely non-opioid analgesia to facilitate early mobilization and shorten hospital stay [1,23,24]. Opioid μ-receptor activation impairs gastrointestinal motility and detrusor function and is strongly associated with postoperative ileus, urinary retention, and refractory nausea and vomiting, directly delaying diet advancement and ambulation [25]. Large health-system analyses have further shown that opioid-related adverse drug events independently prolong length of stay and increase healthcare costs [26,27,28,29]. Within this context, the complete elimination of systemic opioids in our TAP-only protocol—while preserving effective early analgesia—represents a deliberate extension of ERAS objectives in LC.

The extent of analgesia achieved in this study—complete avoidance of IV-PCA, uninterrupted recovery, and rapid return of bowel function—aligns with prior evidence supporting the opioid-minimizing potential of TAP block across abdominal surgeries [30,31,32]. Importantly, universal avoidance of rescue analgesics in our cohort underscores the reproducibility and safety of low-concentration ropivacaine (0.19%) for TAP block, providing sufficient sensory blockade while minimizing concerns regarding local anesthetic systemic toxicity. Network meta-analyses comparing regional techniques for LC have similarly identified subcostal TAP block as one of the most effective approaches for reducing early pain scores and postoperative nausea and vomiting [33,34,35], consistent with our clinical observations.

In contrast, our IV-PCA cohort highlights the inherent instability of opioid-centered analgesia in fast-track LC. Despite protocolized dosing, a substantial proportion of patients developed clinically significant opioid-related adverse effects within the first postoperative hours, frequently leading to early PCA interruption and discontinuation. Notably, stratification by fentanyl dose revealed no clear dose–response relationship, suggesting that opioid toxicity may occur in a threshold-mediated or idiosyncratic manner rather than as a linear function of dose [27,28,29,36,37]. Similar observations have been reported in studies of PONV and opioid intolerance, where susceptibility rather than cumulative dose appears to drive clinical toxicity [38]. These patterns help explain why IV-PCA often fails to provide stable analgesia in ERAS pathways.

From a broader clinical perspective, postoperative pain after LC comprises somatic, visceral, and referred components; however, converging evidence indicates that somatic pain from trocar-site injury predominates during the first 24–48 h and has the greatest functional impact [39,40,41]. By selectively targeting thoracoabdominal sensory nerves innervating these incisions, TAP block addresses the most disabling pain source and facilitates a smooth transition to simple oral analgesics without the need for parenteral rescue. This single-shot TAP only protocol therefore represents a practical, ward-friendly strategy that avoids PCA pumps, intensive monitoring, and increased nursing workload.

In the context of contemporary biliary practice, these findings should also be interpreted against the evolution of preoperative management for gallbladder disease. Modern pathways increasingly incorporate preoperative source control, such as endoscopic retrograde cholangiopancreatography, percutaneous drainage, and guideline-based antibiotic therapy, thereby attenuating the inflammatory burden before LC [17,40,41,42,43]. In such optimized patients, residual early postoperative pain is more likely driven by parietal nociception than uncontrolled visceral inflammation, further supporting the rationale for a regional-first analgesic strategy.

Within this updated clinical context, the present study provides a procedure- and era-specific extension of Bisgaard’s framework [17]. By examining a cohort in which inflammatory pathology was managed according to current standards, we were able to isolate the impact of a single regional technique on early recovery. The single TAP block strategy achieved complete avoidance of IV-PCA, eliminated opioid-induced side effects, and advanced key ERAS milestones by approximately one postoperative day. Although the discharge criteria were implemented within an institutional ERAS framework, the core components align with internationally accepted ERAS recommendations [44], supporting the generalizability of the approach. While visceral or referred pain may not be fully addressed by TAP block alone, future pathology-stratified studies will be important to refine patient selection and determine whether adjunct regional techniques are required in more severe inflammatory phenotypes.

Notwithstanding its retrospective nature, this study provides pragmatic ERAS-related insights by elucidating real-world patterns of IV-PCA failure, such as early interruption and opioid wastage. These findings indicate that a single-shot subcostal TAP block could serve as a practical opioid-sparing analgesic strategy in selected patients undergoing laparoscopic cholecystectomy within ERAS-based care pathways.

## Figures and Tables

**Table 1:** Baseline patient characteristics and standardized mean differences (SMD).

Variable	Total (n = 120)	Group TAP (n = 60)	Group IV-PCA (n = 60)	*p*-Value	SMD
Age (years)	52.70 ± 12.43	51.75 ± 12.73	53.65 ± 12.16	0.405	0.153
Sex				0.175	–
Male	40 (33.3%)	24 (40.0%)	16 (26.7%)		−0.283
Female	80 (66.7%)	36 (60.0%)	44 (73.3%)		
Height (cm)	161.43 ± 8.53	162.83 ± 8.54	160.03 ± 8.35	0.071	−0.332
Weight (kg)	64.45 ± 11.68	66.29 ± 12.38	62.61 ± 10.73	0.084	−0.318
BMI (kg/m^2^)	24.62 ± 3.21	24.81 ± 2.84	24.42 ± 3.56	0.507	−0.121
ASA physical status				0.460	−0.211
ASA I	9 (7.5%)	4 (6.7%)	5 (8.3%)		
ASA II	79 (65.8%)	37 (61.7%)	42 (70.0%)		
ASA III	32 (26.7%)	19 (31.7%)	13 (21.7%)		
Severity of cholecystitis				0.566	−0.139
Grade 1	31 (25.8%)	13 (21.7%)	18 (30.0%)		
Grade 2	75 (62.5%)	40 (66.7%)	35 (58.3%)		
Grade 3	14 (11.7%)	7 (11.7%)	7 (11.7%)		
Preoperative baseline SBP (mmHg)	133.42 ± 15.11	130.30 ± 12.78	136.53 ± 16.66	0.023	0.420
Preoperative baseline DBP (mmHg)	81.38 ± 13.89	76.65 ± 13.48	86.10 ± 12.72	<0.001	0.721
Preoperative baseline heart rate (beats/min)	79.78 ± 18.51	77.70 ± 20.89	81.85 ± 15.67	0.221	0.225
History of PONV				<0.001	−1.206
Yes	32 (26.7%)	32 (53.3%)	0 (0%)		
No	88 (73.3%)	28 (46.7%)	60 (100%)		
Chronic opioid use				0.420	−0.196
Yes	16 (13.3%)	10 (16.7%)	6 (10.0%)		
No	104 (86.7%)	50 (83.3%)	54 (90.0%)		

Values are presented as mean ± standard deviation or number (%). *p*-values were calculated using the independent-samples *t*-test for continuous variables and Pearson’s chi-square test for categorical variables. Severity categories: Grade 1 = chronic cholecystitis without acute inflammatory signs; Grade 2 = acute cholecystitis; Grade 3 = complicated cholecystitis (e.g., empyema, perforation or gangrenous change). History of PONV (postoperative nausea and vomiting) was defined as patient-reported nausea and/or vomiting after previous anesthesia or surgery, documented during pre-anesthetic evaluation. Baseline hemodynamic variables were recorded preoperatively prior to anesthesia induction. Chronic opioid use was defined as daily intake of any opioid analgesic for ≥3 months before surgery. SMD, standardized mean difference. During the study period (January–December 2025), all adult patients (≥18 years) who underwent elective laparoscopic cholecystectomy at our institution were screened for eligibility. Consecutive cases were identified from the institutional surgical registry and anesthesia records. Patients were excluded if they underwent conversion to open surgery, concomitant procedures, or emergency surgery, had incomplete medical records, or had ASA physical status ≥ IV.

**Table 2:** Functional Recovery and Postoperative Analgesic Outcomes.

Outcome Variation Data	TAP (n = 60) Mean ± SD or n (%)	IV-PCA (n = 60) Mean ± SD or n (%)	*p*-Value/Effect Size
Time to first ambulation (h)	3.5 ± 1.2	12.7 ± 4.5	<0.001 (Cohen’s d = 2.83)
Oral intake tolerance time (h)	5.8 ± 2.1	16.3 ± 5.5	<0.001 (d = 2.39)
NRS pain score at 24 h	2.2 ± 1.1	5.8 ± 1.5	<0.001 (d = 2.74)
Rescue analgesic use (0–24 h)	0/60 (0%)	45/60 (75%)	<0.001 (RR = 0.09)
Type of analgesic on POD 1	Acetaminophen 650 mg PO TID only (100%)	Acetaminophen 650 mg PO TID ± NSAID (variable)	—
Discharge within POD 2	60 (100%)	19 (32%)	<0.001 (RR = 3.15)
Length of stay (days)	2.0 (IQR 2, 2)	5.0 (IQR 4, 5)	<0.001 (d = 2.87)

Values are presented as mean ± standard deviation (SD) or number (%). Length of stay (LOS) is presented as median (interquartile range, IQR). Cohen’s d values represent the magnitude of between-group differences, with values ≥ 0.8 generally considered to indicate a large clinical effect. For outcomes summarized using medians, Cohen’s d was calculated based on the underlying continuous data prior to non-parametric summarization.

**Table 3:** Summary of PCA-Related Adverse Events and Interventions.

Outcome Variable	IV-PCA (n = 60) n (%)	TAP (n = 60) n (%)	*p*-Value
Any opioid-related adverse event (OISE)	36 (60.0)	0 (0.0)	<0.001
Headache	14 (23.3)	0 (0.0)	<0.001
Nausea/Vomiting	13 (21.7)	0 (0.0)	<0.001
Dizziness	11 (18.3)	0 (0.0)	<0.001
Additional symptoms (≥2 symptoms)	21 (35.0)	0 (0.0)	<0.001
Any PCA-related intervention required	32 (53.3)	0 (0.0)	<0.001
Temporary PCA clamping (≥1)	32 (53.3)	0 (0.0)	<0.001
Recurrent adverse events	15 (25.0)	0 (0.0)	<0.001

Values are presented as number (%) or mean ± standard deviation (SD). *p*-values were calculated using Fisher’s exact test. The *p*-value for all listed comparisons was statistically significant (*p* < 0.001). No patient in the TAP group experienced opioid-related adverse effects or required any intervention. The item “Premature PCA Discontinuation” specifically refers to the permanent cessation of the IV-PCA regimen, which occurred in 70% of the PCA cohort due to OISEs (primarily nausea and vomiting) during the critical early postoperative period (6–18 h), demonstrating protocol failure.

**Table 4:** Early PCA Disruption and Clinical Management on the Day of Surgery (IV-PCA group, n = 60).

Clinical Event	n (%) or Mean ± SD
PCA clamping on day of surgery (POD 0)	32 (53.3%)
Rescue medication required on POD 0	32 (53.3%)
Antiemetics administered	26 (43.3%)
Non-opioid rescue analgesics *	19 (31.7%)
Recurrent PCA interruption (≥2 events)	15 (25.0%)
PCA permanently discontinued within 48 h	42 (70.0%)
Time from PCA start to first clamping (h)	11.6 ± 4.2
Time from PCA start to declamping/cessation (h)	12.3 ± 5.1
Time from PCA start to final PCA termination (h)	39.4 ± 11.8

* Non-opioid rescue analgesics included ibuprofen, ketorolac, or tramadol, administered at the discretion of the attending physician. Values are presented as number (%) or mean ± standard deviation of Group IV PCA. All events occurred during the early postoperative period, predominantly on the day of surgery. Frequent PCA interruption and early discontinuation reflect intolerance to opioid-based analgesia rather than inadequate analgesic efficacy.

## Data Availability

Data are available upon reasonable request.

## Supplementary Materials

The following supporting information can be downloaded at: https://www.mdpi.com/article/10.3390/jcm15031120/s1, Table S1. Baseline patient characteristics before and after inverse probability of treatment weighting (IPTW); Table S2. Comparison of clinical outcomes after IPTW adjustment; Table S3. Operational definitions and assessment framework.
