# Supplementary material for: Single-Shot Ultrasound-Guided Transversus Abdominis Plane Block Versus Intravenous Patient-Controlled Analgesia for Early Recovery After Laparoscopic Cholecystectomy: A Retrospective Cohort Study
Source: J Clin Med. 2026 Jan 31;15(3):1120. doi: 10.3390/jcm15031120 (PMC12898061; doi:10.3390/jcm15031120)
Supplement: Supplementary file 1 [file jcm-15-01120-s001.zip › jcm-4090508-supplementary.pdf]

Table S1. Baseline patient characteristics before and after inverse probability of treatment weighting (IPTW).

Variable	Unweighted SMD	IPTW-adjusted SMD
Sex	0.28	0.03
Age (years)	0.15	0.04
Height (cm)	0.33	0.05
Weight (kg)	0.32	0.06
Preoperative SBP (mmHg)	0.42	0.07
Preoperative DBP (mmHg)	0.72	0.08
Preoperative HR (beats/min)	0.23	0.05
BMI (kg/m^2)	0.12	0.03
ASA physical status	0.21	0.06
Severity of cholecystitis	0.14	0.04
History of PONV	1.21	0.09
Chronic opioid use	0.20	0.05

Footnote

Standardized mean differences (SMDs) were calculated to assess covariate balance between groups. An SMD <0.1 was considered indicative of adequate balance. IPTW substantially improved balance across all baseline covariates, including history of postoperative nausea and vomiting.

Table S2. Comparison of clinical outcomes after IPTW adjustment.

Outcome	TAP block (IPTW-adjusted)	IV-PCA (IPTW-adjusted)	Effect estimate
Length of stay (days), mean ± SD	2.1 ± 0.4	4.8 ± 0.7	Mean difference −2.7 days
Discharge by POD2, n (%)	58 (96.7%)	20 (33.3%)	OR 12.4
Rescue analgesic use (0–24 h), n (%)	1 (1.7%)	43 (71.7%)	OR 0.02
Any opioid-related adverse event, n (%)	0 (0.0%)	35 (58.3%)	OR <0.01

Footnote

Outcome comparisons were performed using IPTW-weighted regression models. Odds ratios (ORs) were estimated using weighted logistic regression. Results remained directionally consistent with unadjusted analyses, confirming robustness of the findings after adjustment for confounding by indication.

Table S3. Operational definitions and assessment framework.

Domain	Definition	Assessment method
Opioid-related adverse events	Nausea, vomiting, dizziness, headache, urinary retention, delayed flatus	Retrospective extraction from standardized postoperative nursing records
Timing of adverse event assessment	From ward arrival to POD2	Routine nursing documentation
Early postoperative period (<1 h)	No adverse events observed in either group	PACU and immediate ward records
Intraoperative opioid exposure	Standardized general anesthesia protocol	Anesthesia record (agent and total dose)
Discharge readiness	Adequate pain control, oral intake tolerance, ambulation, stable vital signs, no complications	Institutional ERAS-based checklist
Outcome adjudication	Ward nurses and attending physicians	Routine clinical documentation

Footnote

Adverse events and discharge readiness were assessed using identical documentation frameworks in both groups as part of routine postoperative care, minimizing detection bias. Discharge readiness criteria were based on institutional ERAS-aligned protocols.
